# Dr. A. McFarland

**Published:** 1870-09

**Authors:** 


***Dr. A. McFarland,***

Late Superintendent of the State Hospital for the Insane at Jacksonville, presents himself in an able letter to the electors of Morgan county for a seat in the lower house in the next legislature. The reasons which impel him to the step are lucidly set forth, and, independently of politics, of which we know little and care less, we hope our Morgan county fellow-citizens will honor themselves by sending this high-minded, intelligent gentleman to represent them. Scarcely another man in the State is more competent to deal with the great interests involved in our eleemosynary institutions. It appears that an attack, intended in the first instance to be personally upon Dr. McF., in the event became the prime cause of placing upon the statute book of Illinois, one of the most atrocious pieces of legislation that ever disgraced a deliberative assembly. Practically, we know it to be oppressive and absurd in the last degree.

A brief extract from Dr. McF.'s letter sets forth the fact clearly, and we do not need to apologize for introducing it.

"Among the base measures used by an unscrupulous ring, was a simultaneous attack on all State interests of Jacksonville. The attack got an especial point as regards myself from the appearance on the stage, at this particular time, of an insane woman, whose rebuked free-love advances gave to her malignity all the venom proverbially attributed to the 'woman scorned.' Of all that followed for a year afterwards, at the cost, to the State, of many thousands of dollars, with no result except to enable an ambitious politician, of little capacity and less principle, to figure for his brief hour on the stage, I make no mention—the whole being too purely personal, and only touched upon here as prefatory of the particular matter I wish to introduce.

"Among the devices conceived to annoy and punish the individual particularly aimed at, was an enactment, speciously entitled: 'An Act for the Protection of Personal Liberty,' clearly intended as a stinging insult to the Superintendent of the State Hospital; imputing to him a disposition to commit a high crime against the liberty of the innocent unfortunate, and appending the most degrading penalties known to the law for the offense. An act to prevent John Brown, of a particular city, street and number, from stealing sheep, could be no more justly offensive to the John Brown indicated than is this enactment to myself. But the personal affront, attempted in the passage of the act, bears no proportion whatever to the wide-spread and cruel wrong it has inflicted on the class it is ostensibly intended to benefit. Its first effect was to draw from the quiet of their apartments nearly all the inmates of the State Hospital, and subject them to the mockery of a new inquisition—a measure full of disaster, but for the rare good sense and discretion of a Morgan county court and jury. Since that period no individual in Illinois, whatever may have been the circumstances of sex, age, physical health or residence, who has needed treatment at the institution, but has had to pass the harsh ordeal laid down in this most singular statute. It converts every unfortunate insane person into a DEFENDANT IN A PROSECUTION, in which the dearest friends of the party are, in the necessity of the case, converted into apparent enemies. It often takes the invalid from the bed of sickness, to be transported, perhaps miles in an opposite direction from the institution, to a county seat, there to be adjudged by the ignorant hangers-on of a court house, who must hear, prior to their decision, the secrets of the family and the sick-room laid bare as the accidents of the case may determine. What should be decided by a county judge, in the privacy of his chambers, and upon the certificates of medical men, is made to depend upon the haphazard opinions and caprices of men grossly ignorant of the subject before them, the obvious effect of which frequently is to defeat the most kindly intentions of friends, and consign to hopeless insanity many whom a more ready access to the institution would have restored to reason and society. This enactment has met the derision, and received the protest of every philanthropic mind throughout the land. Persons of any sensibility who have the means, evade it by transporting insane friends to distant private institutions. Within a year, the ecclesiastical head of a great religious body, and also a member of the convention chosen to frame a new constitution for the State, have been carried out of the State rather than be submitted to so odious an inquisition. The imagined wrong it was pretendedly set up to correct, thus affords a pretext for a greater possible wrong, in this constant abduction."

